# The Use of Human Serum Samples to Study Malignant Transformation: A Pilot Study

**DOI:** 10.3390/cells10102670

**Published:** 2021-10-06

**Authors:** Andreana N. Holowatyj, Biljana Gigic, Christy A. Warby, Jennifer Ose, Tengda Lin, Petra Schrotz-King, Cornelia M. Ulrich, Jamie J. Bernard

**Affiliations:** 1 Department of Medicine, Vanderbilt University Medical Center, Nashville, TN 37232, USA; 2 Vanderbilt-Ingram Cancer Center, Nashville, TN 37232, USA; 3 Huntsman Cancer Institute, Salt Lake City, UT 84112, USA; Christy.Warby@hci.utah.edu (C.A.W.); Jennifer.ose@hci.utah.edu (J.O.); tengda.lin@hci.utah.edu (T.L.); Neli.ulrich@hci.utah.edu (C.M.U.); 4 Department of Population Health Sciences, University of Utah, Salt Lake City, UT 84112, USA; 5 Department of General, Visceral and Transplantation Surgery, Heidelberg University Hospital, 69120 Heidelberg, Germany; Biljana.Gigic@med.uni-heidelberg.de; 6 German Cancer Research Center (DKFZ), 69120 Heidelberg, Germany; petra.schrotz-king@nct-heidelberg.de; 7 National Center for Tumor Diseases (NCT), 69120 Heidelberg, Germany; 8 Department of Pharmacology and Toxicology, Michigan State University, East Lansing, MI 48823, USA

**Keywords:** FGF2, body mass index, obesity, overweight, body fatness

## Abstract

Obesity and excess adiposity account for approximately 20% of all cancer cases; however, biomarkers of risk remain to be elucidated. While fibroblast growth factor-2 (FGF2) is emerging as an attractive candidate biomarker for visceral adipose tissue mass, the role of circulating FGF2 in malignant transformation remains unknown. Moreover, functional assays for biomarker discovery are limited. We sought to determine if human serum could stimulate the 3D growth of a non-tumorigenic cell line. This type of anchorage-independent 3D growth in soft agar is a surrogate marker for acquired tumorigenicity of cell lines. We found that human serum from cancer-free men and women has the potential to stimulate growth in soft agar of non-tumorigenic epithelial JB6 P^+^ cells. We examined circulating levels of FGF2 in humans in malignant transformation in vitro in a pilot study of *n* = 33 men and women. Serum FGF2 levels were not associated with colony formation in epithelial cells (*r* = 0.05, *p* = 0.80); however, a fibroblast growth factor receptor-1 (FGFR1) selective inhibitor significantly blocked serum-stimulated transformation, suggesting that FGF2 activation of FGFR1 may be necessary, but not sufficient for the transforming effects of human serum. This pilot study indicates that the FGF2/FGFR1 axis plays a role in JB6 P^+^ malignant transformation and describes an assay to determine critical serum factors that have the potential to promote tumorigenesis.

## 1. Introduction

Epidemiologic and experimental studies have linked obesity, particularly abdominal obesity, as a critical risk factor to thirteen different cancer types [1]. Adipose tissue functions as a dynamic endocrine organ that secretes numerous tumor-promoting factors involved in the regulation of hormonal and inflammatory pathways [2]. Body mass index (BMI) is the most common anthropometric measure of obesity and is used to stratify individuals at high risk for metabolic disorders [3]. However, BMI does not discriminate between adipose tissue and muscle, nor does it directly assess regional adiposity [4]. Recent findings from Haffa et al. have further shed light that BMI poorly reflects fat mass-associated changes in visceral adipose tissue (VAT) [5]. In contrast to subcutaneous adipose tissue (SAT), we and others have demonstrated that excessive visceral adipose tissue (VAT) is more strongly associated with metabolic perturbations and inflammation [5,6,7,8]. Our prior studies also suggest that VAT secretion of fibroblast growth factor-2 (FGF2) is associated with malignant transformation [9,10]. However, it is unknown if human serum-derived FGF2 stimulate malignant transformation in vitro. Moreover, advances in biomarker discovery are limited due to the lack of functional assays that test the effects of human serum of specific biomarkers within serum on malignant transformation. Therefore, we sought to determine if human serum could be used to stimulate the 3D growth of a non-tumorigenic cell line. Growth in soft agar is a type of 3D anchorage-independent growth often used as a surrogate marker for malignant transformation. We found that human serum from cancer-free men and women has the potential to stimulate growth in soft agar and this assay can be used to determine critical serum factors that may promote tumorigenesis.

## 2. Materials and Methods

Human serum (*n* = 8) was purchased from Spectrum Health Universal Biorepository (SHUB) and analyzed for the ability to stimulate JB6 P^+^ cell growth in soft agar in the presence or absence fibroblast growth factor receptor-1 (FGFR1) inhibitor. The fibroblast growth factor receptor-1 (FGFR1) inhibitor, PD166866, was purchased from SelleckChem, growth factors were purchased from R&D Systems (insulin-like growth factor-1 (IGF1) and FGF2) and estradiol was purchased from Sigma-Aldrich. The PD166866 concentration used herein was based upon our previously published dose–response studies showing that 2.5 μM blocks FGF2-stimulated malignant transformation in JB6 P^+^ cells [11].

The study population for which FGF2 was measured includes *n* = 33 individuals from the PRÄVENT Study, a cohort of cancer-free men and women aged 18 to 89 years, recruited between July 2013 and December 2014 in Heidelberg, Germany. All PRÄVENT participants consented to participate in the study, and samples were collected during a recruitment visit [12]. This study was approved by the ethics committee at the University of Heidelberg. All study participants provided written informed consent.

Serum-based assays for biomarkers of inflammation have previously been established on the Mesoscale Discovery Platform (MSD, Rockville, MD, USA) and described [12]. Growth in soft agar is a surrogate marker of malignant transformation and is quantified by colony count. JB6 P^+^ (mouse skin epithelial) cells were plated in soft agar with 10 µL of human serum and cultured for 10 days. The colony formation assay was performed and quantified as previously described [13]. Samples were blinded and run in duplicate and then averaged to quantify % colony formation. Colonies were counted using the Cytation 3 imaging reader from Biotek and Gen5 3.04 software. Seven pictures were taken every 100 microns and superimposed together by the zprojection function.

Distribution of study population characteristics by sex were summarized by frequency and compared using chi-square or *t*-tests. Pearson correlation coefficients were used to assess the correlation between FGF2 levels and colony formation (%). Stratified analyses were performed by sex and age at blood draw (age younger than 65 years vs. 65+ years). All analyses were conducted in SAS version 9.4 statistical software (SAS Institute; Cary, NC, USA). All tests were two-sided, with *p* ≤ 0.05 considered to be statistically significant. Graphs were generated using Graphpad Prism 7 software.

## 3. Results

**Figure 1:**
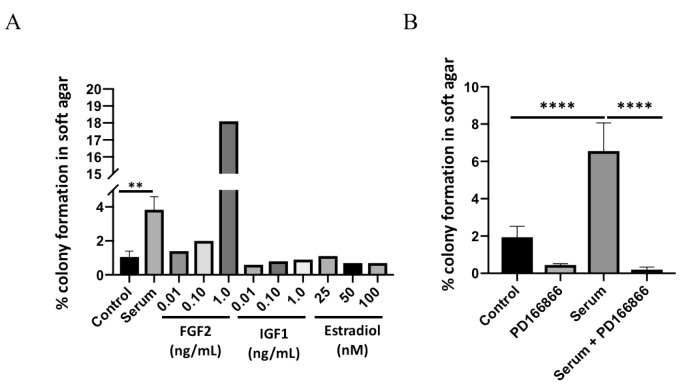
Human serum stimulates FGFR1-dependent colony formation. Serum significantly stimulates JB6 P^+^ colony formation (**A**). Control (no treatment) and serum treatments were analyzed by a two-tailed *t* test (** *p* < 0.01). PD166866 (2.5 μM) significantly blocks serum-stimulated colony formation (**B**). Each treatment group was analyzed by one-way ANOVA with multiple comparisons (**** *p* < 0.0001).

Only recombinant FGF2 protein stimulated colony formation in JB6 P^+^ cells; no stimulation was observed with IGF1 and estradiol. (Figure 1A). Insulin like growth factor-1 (IGF1) and estradiol were used as comparative tumor promoters to FGF2. The stimulatory concentration of FGF2 on growth in soft agar (>0.1 ng/mL) is similar to what has been reported for physiological serum concentrations in obesity [14]. JB6 P^+^ cells cultured with human serum demonstrated a significantly higher percentage of cells growing in soft agar (mean = 3.8%) compared with the untreated control cells (mean = 1%; Figure 1B). To determine if the stimulatory effect of serum was due to FGFR1 signaling, JB6 P^+^ cells treated with serum were cultured in the presence of a selective FGFR1 inhibitor, PD166866. PD166866 significantly inhibited serum-stimulated growth in soft agar (Figure 1B).

**Figure 2:**
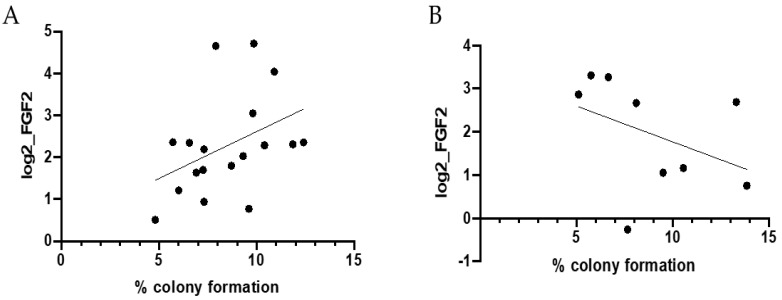
Correlation between human FGF2 levels and colony formation. Pearson correlations between human serum FGF2 levels and colony formation (%) among cancer-free (**A**) females age younger than 65 years at blood draw (*r* = 0.40, *p* = 0.098) and (**B**) males age younger than 65 years at blood draw (*r* = −0.40, *p* = 0.28). Individuals age 65 years and older were not included in stratified analyses due to limited sample size (*n* = 4 females age 65+ years at blood draw; *n* = 2 males age 65+ years at blood draw).

Further studies were performed to examine a cancer-free cohort of subjects for serum FGF2 and activity on colony formation. The baseline characteristics of the study population by sex are presented in Table 1. Mean age at blood draw was 55.2 years (standard deviation [std], 13.2 years). Two-thirds of the patients (*n* = 21 of 33; 63.6%) were overweight or obese, with a mean BMI classified as overweight (26.4 kg/m^2^; std, 5.1 kg/m^2^; Table 1). By sex, no significant differences in age or body mass index were noted in this study population (*p* ≥ 0.60). Serum FGF2 levels were not associated with colony transformation in epithelial cells (*r* = 0.05, *p* = 0.80; data not shown). Stratification by sex and age revealed patterns between human serum FGF2 levels and colony transformation in vitro (Figure 2). Among women age younger than 65 years, serum FGF2 levels were positively correlated with colony transformation, with marginal statistical significance (r = 0.40, *p* = 0.098; Figure 2A). Inverse associations between serum FGF2 levels with in vitro colony transformation were noted among males age younger than 65 years, although these findings were not statistically significant (*r* = −0.40, *p* = 0.28; Figure 2B).

## 4. Discussion

Current estimates suggest that obesity and excess adiposity account for approximately one-fifth of all cancer cases [1]. Previous studies have implicated FGF2 in the obesity–cancer axis, as VAT levels of FGF2 were positively correlated with the capacity of VAT to stimulate mammary and skin epithelial cell growth, and circulating FGF2 levels were associated with overall adipose tissue mass in humans [15].

Herein, our feasibility study did not observe patterns between human serum FGF2 levels and colony transformation in vitro, although differential effects emerged when the cases were stratified by sex. However, a novel aspect of this study is the methodological approach that utilizes serum from a cohort of healthy individuals to bridge the laboratory setting with clinical studies and is therefore closer to clinical application.

Our previous work has demonstrated that inhibitors of fibroblast growth factor receptor-1 (FGFR1), the primary receptor tyrosine kinase (RTK) that the FGF2 ligand binds, block the VAT-induced colony formation—suggesting that the FGF2/FGFR1 axis may be critical in the context of adiposity-associated malignant transformation [9,10]. Additionally, our prior mouse studies demonstrated that circulating FGF2 was associated with both visceral adiposity and ultraviolet radiation-induced skin cancer [10,16] and an epidemiological study demonstrated that circulating FGF2 levels in humans are associated with overall adipose tissue mass [15]. While FGF1 is present in white adipose tissue and activates FGFR1, adipose tissue-derived FGF1 does not enter the circulation [17]. To date, it is unknown whether or not sera FGF2 is associated with VAT mass in humans. While BMI data were obtained from this cohort, adipose tissue mass and regional adiposity was not available for assessment. Therefore, further studies are warranted to examine the relationship between circulating FGF2 and visceral adiposity.

Colony formation assays suggest a sex-specific role for circulating FGF2 in cellular transformation and that FGF2 activation of FGFR1 may be necessary but not sufficient for cellular transformation. Serum FGF2 and colony transformation were marginally significantly correlated among healthy, cancer-free females aged less than 65 years. Intriguingly, these findings suggest that estrogen may play a role in the response to FGF2; however, estradiol alone did not have an effect on colony formation in JB6 P^+^ cells. To our knowledge, there have been no reports that estradiol stimulates JB6 P^+^ cells transformation. In fact, oppositely, epidemiology and preclinical studies suggest that estrogen is protective in nonmelanoma skin cancers [18,19,20,21,22]. However, this does not preclude estrogen from interacting with other growth factor signaling pathways to have a pro-carcinogenic effect. For example, estrogen can also activate Akt and Erk signaling epidermal keratinocytes [23], downstream mediators of fibroblast growth factor receptor (FGFR) activation. Moreover, FGFR and estrogen receptor pathways are activated in models of lung and breast cancer, but how these pathways may interact during malignant transformation remains unknown. Given the inverse associations between human FGF2 levels in serum and in vitro colony transformation we observed in our pilot study, additional investigations are needed to understand the potential sex-specific role for FGF2 in malignant transformation. For these types of studies, we would utilize estradiol-responsive MCF-10A human mammary epithelial cells.

While the use of human sera from healthy individuals to assess in vitro colony transformation is a strength of this study, we also acknowledge the limitations of this work. Measures of body fatness, including visceral fat area and subcutaneous fat area, were not collected in this pilot study, along with the limited sample size of this cohort. Moreover, we only tested the effects of serum on one cell line. Our findings suggest that mechanisms of FGF2 cellular transformation may differ by sex and that FGF2 activation is necessary but not efficient for cellular transformation. Further investigations into the sex-specific mechanisms of FGF2 in in vitro and in vivo cellular transformation in multiple models are warranted in larger and more diverse populations, as it remains unclear as to whether FGF2 levels in circulating blood are associated with visceral adiposity in humans.

## Figures and Tables

**Table 1:** Clinical and demographic characteristics listed by sex among individuals included in this pilot study.

	Total		Female		Male		
*Characteristic*	N	%	N	%	N	%	*p*
**Total**	33	100.0	22	66.7	11	33.3	
**Age**							1.0
<65 Years	27	81.8	18	81.8	9	81.8	
65+ Years	6	18.2	4	18.2	2	18.2	
Mean, Years (std)	55.2	(13.2)	56.0	(13.7)	53.5	(12.6)	0.62
**Body Mass Index** (BMI, kg/m^2^)							0.71
Underweight/Normoweight, <25	12	36.4	9	40.9	3	27.3	
Overweight, 25–29.99	15	45.5	9	40.9	6	54.5	
Obese, 30+	6	18.2	4	18.2	2	18.2	
Mean, kg/m^2^ (std)	26.4	(5.1)	26.3	(6.0)	26.6	(2.6)	0.87

## Data Availability

The data are available upon request from the corresponding author.

## References

[1] Lauby-Secretan B., Scoccianti C., Loomis D., Grosse Y., Bianchini F., Straif K. (2016). International Agency for Research on Cancer Handbook Working, G. Body Fatness and Cancer—Viewpoint of the IARC Working Group. N. Engl. J. Med..

[2] Ulrich C.M., Himbert C., Holowatyj A.N., Hursting S.D. (2018). Energy balance and gastrointestinal cancer: Risk, interventions, outcomes and mechanisms. Nat. Rev. Gastroenterol. Hepatol..

[3] Caan B.J., Cespedes Feliciano E.M., Kroenke C.H. (2018). The Importance of Body Composition in Explaining the Overweight Paradox in Cancer-Counterpoint. Cancer Res..

[4] Stevens J., McClain J.E., Truesdale K.P. (2008). Selection of measures in epidemiologic studies of the consequences of obesity. Int. J. Obes..

[5] Haffa M., Lin T., Holowatyj A.N., Kratz M., Toth R., Benner A., Gigic B., Habermann N., Schrotz-King P., Bohm J. (2019). Transcriptome profiling of adipose tissue reveals depot-specific metabolic alterations among colorectal cancer patients. J. Clin. Endocrinol. Metab..

[6] Ose J., Holowatyj A.N., Nattenmuller J., Gigic B., Lin T., Himbert C., Habermann N., Achaintre D., Scalbert A., Keski-Rahkonen P. (2020). Metabolomics profiling of visceral and abdominal subcutaneous adipose tissue in colorectal cancer patients: Results from the ColoCare study. Cancer Causes Control.

[7] Holowatyj A.N., Haffa M., Lin T., Scherer D., Gigic B., Ose J., Warby C.A., Himbert C., Abbenhardt-Martin C., Achaintre D. (2020). Multi-omics analysis reveals adipose-tumor crosstalk in colorectal cancer patients. Cancer Prev. Res..

[8] Himbert C., Ose J., Nattenmuller J., Warby C.A., Holowatyj A.N., Bohm J., Lin T., Haffa M., Gigic B., Hardikar S. (2019). Body Fatness, Adipose Tissue Compartments, and Biomarkers of Inflammation and Angiogenesis in Colorectal Cancer: The ColoCare Study. Cancer Epidemiol. Biomark. Prev..

[9] Benham V., Chakraborty D., Bullard B., Bernard J.J. (2018). A role for FGF2 in visceral adiposity-associated mammary epithelial transformation. Adipocyte.

[10] Chakraborty D., Benham V., Bullard B., Kearney T., Hsia H.C., Gibbon D., Demireva E.Y., Lunt S.Y., Bernard J.J. (2017). Fibroblast growth factor receptor is a mechanistic link between visceral adiposity and cancer. Oncogene.

[11] Benham V., Bullard B., Dexheimer T.S., Bernard M.P., Neubig R.R., Liby K.T., Bernard J.J. (2019). Identifying chemopreventive agents for obesity-associated cancers using an efficient, 3D high-throughput transformation assay. Sci. Rep..

[12] Holowatyj A.N., Gigic B., Herpel E., Scalbert A., Schneider M., Ulrich C.M., Metabo C.C.C.C., ColoCare S. (2019). Distinct Molecular Phenotype of Sporadic Colorectal Cancers Among Young Patients Based on Multiomics Analysis. Gastroenterology.

[13] Chakraborty D., Benham V., Bernard J.J. (2017). Elucidating the role of adipose tissue secreted factors in malignant transformation. Adipocyte.

[14] Larsson A., Skoldenberg E., Ericson H. (2002). Serum and plasma levels of FGF-2 and VEGF in healthy blood donors. Angiogenesis.

[15] Hao R.H., Guo Y., Dong S.S., Weng G.Z., Yan H., Zhu D.L., Chen X.F., Chen J.B., Yang T.L. (2016). Associations of Plasma FGF2 Levels and Polymorphisms in the FGF2 Gene with Obesity Phenotypes in Han Chinese Population. Sci. Rep..

[16] Lu Y.P., Lou Y.R., Bernard J.J., Peng Q.Y., Li T., Lin Y., Shih W.J., Nghiem P., Shapses S., Wagner G.C. (2012). Surgical removal of the parametrial fat pads stimulates apoptosis and inhibits UVB-induced carcinogenesis in mice fed a high-fat diet. Proc. Natl. Acad. Sci. USA.

[17] Mejhert N., Galitzky J., Pettersson A.T., Bambace C., Blomqvist L., Bouloumie A., Frayn K.N., Dahlman I., Arner P., Ryden M. (2010). Mapping of the fibroblast growth factors in human white adipose tissue. J. Clin. Endocrinol. Metab..

[18] Yao P.L., Gonzalez F.J., Peters J.M. (2014). Targeting estrogen receptor-beta for the prevention of nonmelanoma skin cancer. Cancer Prev. Res..

[19] Mancuso M., Gallo D., Leonardi S., Pierdomenico M., Pasquali E., De Stefano I., Rebessi S., Tanori M., Scambia G., Di Majo V. (2009). Modulation of basal and squamous cell carcinoma by endogenous estrogen in mouse models of skin cancer. Carcinogenesis.

[20] Staples M.P., Elwood M., Burton R.C., Williams J.L., Marks R., Giles G.G. (2006). Non-melanoma skin cancer in Australia: The 2002 national survey and trends since 1985. Med. J. Aust..

[21] Hayes R.C., Leonfellner S., Pilgrim W., Liu J., Keeling D.N. (2007). Incidence of nonmelanoma skin cancer in New Brunswick, Canada, 1992 to 2001. J. Cutan Med. Surg..

[22] Ramachandran S., Fryer A.A., Lovatt T.J., Smith A.G., Lear J.T., Jones P.W., Strange R.C. (2003). Combined effects of gender, skin type and polymorphic genes on clinical phenotype: Use of rate of increase in numbers of basal cell carcinomas as a model system. Cancer Lett..

[23] Zhou T., Yang Z., Chen Y., Chen Y., Huang Z., You B., Peng Y., Chen J. (2016). Estrogen Accelerates Cutaneous Wound Healing by Promoting Proliferation of Epidermal Keratinocytes via Erk/Akt Signaling Pathway. Cell. Physiol. Biochem..

